# Beyond subsets: single cell transcriptomics reveals multidimensional regulation of iNKT cells cross tissues and species

**DOI:** 10.3389/fimmu.2026.1874403

**Published:** 2026-07-15

**Authors:** Meng Zhao, Lihua Wu

**Affiliations:** 1 Arthritis and Clinical Immunology Research Program, Oklahoma Medical Research Foundation, Oklahoma, OK, United States; 2 Department of Microbiology and Immunology, University of Oklahoma Health Sciences Center, Oklahoma, OK, United States; 3 Stephenson Cancer Center, University of Oklahoma Health Sciences Center, Oklahoma, OK, United States

**Keywords:** differentiation, human, iNKT (invariant natural killer T cell), mouse, periphery, scRNAseq, thymus

## Abstract

Invariant natural killer T (iNKT) cells are innate-like lymphocytes that rapidly respond to lipid antigens presented by CD1d or to inflammatory cytokines and influence diverse immune responses. Much of our current understanding of iNKT cell biology derives from murine studies, which established a framework of thymic differentiation into NKT1, NKT2, and NKT17 subsets. Recent single-cell RNA sequencing (scRNAseq) studies have substantially expanded this view by revealing non-linear developmental trajectories, early epigenomic priming, and a multipotent recent thymic emigrant population that continues to differentiate after thymic egress. In peripheral tissues, iNKT cells undergo extensive remodeling driven by local environmental cues and antigen exposure, giving rise to regulatory, and effector states not observed in the thymus. At the same time, emerging human studies reveal principles that differ from those described in mice. Human iNKT cells exhibit a blended type 1/type 17 transcriptional program, limited evidence for NKT2-like populations, and functionally distinct CD4+, double-negative (DN), CD8αα+, and terminal effector-like subsets. Comparative analyses across species further suggest that differences in thymic selection, transcription factor networks, and peripheral maturation contribute to divergent patterns of iNKT cell specialization. Together, these findings support a revised view of iNKT cells as dynamic and context-dependent transcriptional states shaped by developmental history, tissue environment, antigen exposure, and species-specific regulatory programs.

## Introduction

Invariant natural killer T (iNKT) cells are a conserved population of innate-like T lymphocytes that occupy a unique position at the interface of innate and adaptive immunity. Unlike conventional αβ T cells, iNKT cells express a semi-invariant T cell receptor (TCR), composed of an invariant Va14-Ja18 chain in mice and a homologous Va24-Ja18 chain in humans paired with a restricted set of TCRb chains. This receptor recognizes lipid antigens presented by the non-polymorphic MHC class I-like molecule CD1d, enabling rapid activation and the production of large quantities of cytokines shortly after stimulation (1). Through interactions with dendritic cells, macrophages, B cells, NK cells, and conventional T cells, iNKT cells influence diverse processes including antimicrobial immunity, tumor surveillance, autoimmunity, humoral responses, and tissue homeostasis. These properties also made iNKT cells attractive candidates for off-the-shelf immunotherapies (2).

**Figure 1:**
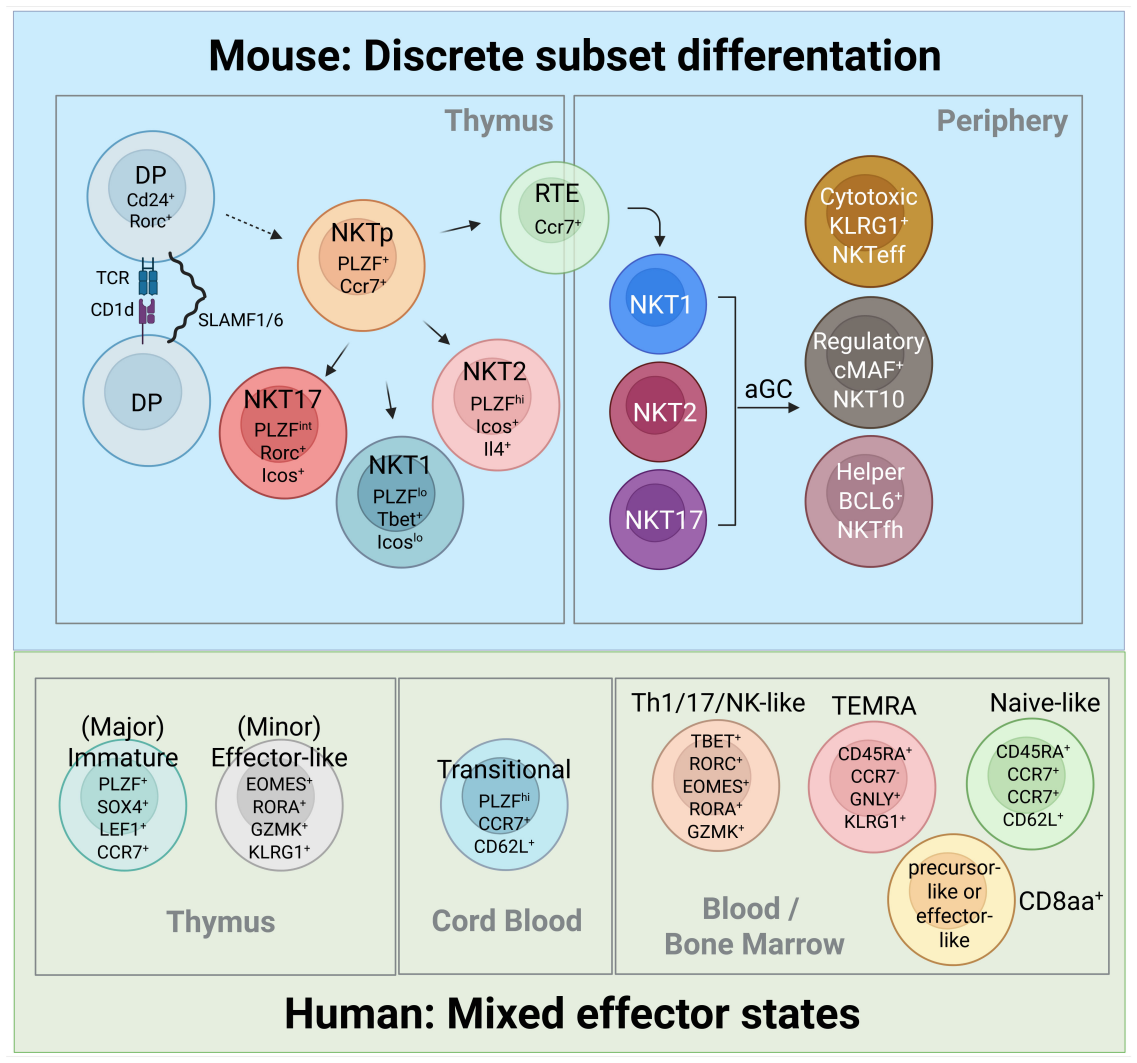
scRNAseq analyses reveal species-specific organization of iNKT cell differentiation. (Top) In mice, iNKT cell thymic selection drives commitment to discrete NKT1, NKT2, and NKT17 effector subsets that are maintained in the periphery and can further diversify upon activation. (Bottom) In humans, iNKT cells follow a predominantly linear differentiation trajectory without discrete subset segregation. Thymic iNKT cells are mostly immature, with a minor effector-like fraction. Cord blood cells occupy a transitional state, while adult peripheral blood and bone marrow are dominated by a mixed Th1/17/NK-like effector program, alongside minor TEMRA-like and naïve-like populations. CD8^+^ iNKT cells predominantly express CD8αα homodimers and span precursor-like and effector transcriptional states.

Most mechanistic insight into iNKT cell biology has been derived from murine studies. In mice, iNKT cells develop through a series of defined stages following selection by CD1d-expressing double positive thymocytes. Early studies described a linear maturation process based on CD24, CD44 and NK1.1 expression, which was later complemented by a functional classification into three major subsets-NKT1, NKT2 and NKT17, characterized by expression of Tbet, PLZF/GATA3, or RORgt, and preferential production of IFNg, IL-4, or IL-17 respectively (3–5) (**Figure 1**). Subsequent work has demonstrated that subset differentiation can emerge at intermediate stages, and distinct transcriptional programs may be initiated prior to terminal maturation, indicating partial overlap between these models (6). Furthermore, iNKT cells are widely distributed across peripheral tissues (7, 8). It remains unclear to what extent peripheral iNKT cell diversity reflects the seeding of fully differentiated thymic subsets instead of ongoing differentiation and remodeling after thymic egress (9).

Single-cell RNA sequencing (scRNAseq) has refined this framework by revealing continuous differentiation trajectories, transitional populations, and tissue-specific transcriptional programs (6, 10–16). These data challenged the rigid subset model and highlighted the importance of post-thymic remodeling. Parallel advances in human and comparative transcriptomic studies have further expanded our understanding of iNKT cell biology by identifying both conserved and species-specific features of differentiation and effector function (12, 13, 15, 16). In this review, we discuss how single-cell studies have refined current models of thymic development, uncovered mechanisms of tissue-specific remodeling, and revealed important similarities and differences in iNKT cell organization across species.

## Thymic foundations: what single cell analyses revealed

In an integrated scRNAseq and scATACseq analysis of stage-sorted thymic iNKT cells, Wang et al. identified heterogeneity within the ST0 compartment and proposed two potential developmental trajectories (17). These include a DP to CD4SP route associated with increased *Zbtb16* accessibility and a bias toward iNKT2 differentiation, and a DP to DN route associated with *Rorc* accessibility and features linked to iNKT17 differentiation. Notably, signatures associated with iNKT1 cells were not detected at the ST0 stage, suggesting that acquisition of the iNKT1 program may occur later. An alternative developmental pathway has previously been reported in which a small population of CD4^–^ NKT1 cells can arise directly from double-negative (DN) thymocytes, bypassing the conventional CD4^+^ intermediate stage (18). While these findings suggest subset bias may arise shortly after positive selection, the interpretation of early commitment at ST0 will benefit from further investigation. Additionally, this study also identified transcriptional regulators, including Cbfβ, as important contributors to early iNKT cell development. Extending these observations to humans, Loh et al. reported that most postnatal thymic iNKT cells resemble naïve conventional T cells transcriptionally, characterized by expression of CCR9, TOX, and SATB1 at early stages and CCR7 at later stages. This naïve-like state was confirmed by flow cytometry (CD161-EOMES-GZMK-) and was shared across multiple innate-like T (Tinn, iNKT, MAIT, gdT) cell populations. Notably, only a minority Tinn displayed an effector signature (15).

Cross-species analyses revealed reduced thymic diversification outside of mice. In pigs and humans, iNKT cells exhibit more linear developmental trajectories with predominantly immature phenotypes and limited resolution of canonical subsets (12, 13, 19). Human thymic iNKT cells are enriched for SOX4, SATB1, LEF1, TOX2 and ITM2A, genes associated with early precursors and ST0 cells in mice and display high expression of ZBTB16/PLZF but minimal TBX21 or RORC (12). Notably, SOX4 regulon activity shows the strongest negative correlation with pseudotime across the entire differentiation trajectory, highlighting its specific role in earliest thymic stages. Comparison with peripheral blood datasets indicate that the more differentiated subset is shared between thymus and periphery, while the most immature precursor population is largely thymus restricted (12). Human thymic iNKT cells that do acquire an effector phenotype converge on a mixed type1/type17 transcriptional state, co-expressing EOMES and GZMK together with RORA and CCR6, instead of TBX21 and RORC as in mice (15). The reduced thymic diversification observed in humans may reflect species-specific differences in thymic selection and co-stimulatory signaling (15). In mice, iNKT cell maturation depends on homotypic interactions between DP thymocytes mediated by SLAMF1 and SLAMF6, which drive PLZF upregulation (20). In humans, however, SLAMF6 is not detectable on DP thymocytes. In addition, whereas murine medullary thymic epithelial cells (mTECs) express CD1d, human mTECs do not, restricting thymic CD1d mediated antigen presentation primarily to cortical thymic epithelial cells (cTECs) and DP thymocytes. Together, these differences might contribute to the incomplete effector iNKT cell differentiation in the thymus, with further maturation likely deferred to peripheral tissues (15).

Early studies, including cytokine reporter and fate-mapping approaches, established NKT2 cells as a distinct functional subset characterized by high PLZF expression and constitutive IL-4 production (4, 9, 21). However, recent scRNAseq studies have consistently identified NKT2 populations as transcriptionally related to precursor-like states (10), with one study reporting substantial overlap between NKT2 and iNKT precursor (NKTp) signatures (22), while another resolving NKT2 into distinct subgroups (NKT2a and NKT2b) (23). In both cases, trajectory inference places NKT2-like populations at an intermediate position along developmental paths leading toward NKT1 and NKT17 fates, and transcriptome reveals co-expression of lineage associated programs within these cells (10, 23). These findings suggest that NKT2 cells represent a heterogeneous population that includes cells with developmental plasticity. At the same time, trajectory-based analyses are limited by their reliance on computational inference and snapshot data, and do not exclude the existence of functionally stable IL-4 producing NKT2 cells *in vivo*. A first scRNAseq analysis of human thymic iNKT cells identified an immature population enriched for a murine NKT2 signature, whereas more differentiated populations display NKT1-like features (13). However, these NKT2-like cells also expressed developmental regulators during immature precursor stage rather than a distinct effector lineage. Consistent with this interpretation, subsequent analyses failed to detect a discrete NKT2 population in humans expressing IL-4 or IL-13 transcripts (15).

An additional conserved feature is the presence of iNKT populations enriched for interferon-stimulated genes (ISGs) in pigs (19), paralleling ISG-high iNKT populations reported in murine datasets (14, 22, 23). Interestingly, disruption of type I interferon signaling preferentially impairs iNKT1 and iNKT17 populations, supporting a role for interferon signaling in shaping subset differentiation in mice (23). In humans, an analogous ISG-associated cluster was identified in both thymic iNKT and MAIT cells, marked by MX1, IFI6, and IFI44L (15). ISG-associated transcriptional programs were similarly observed in peripheral iNKT cells (12). The thymic effector Tinn cells display tissue specific gene expression profiles distinct from circulating blood cells, arguing against the interpretation that ISG-high thymic cells are simply blood derived contaminants (15).

## Post-thymic maturation: recent thymic emigrants as a transition state

A key implication of recent work is that thymic egress does not release fully differentiated iNKT effector subsets. Instead, mature thymic NKT1, NKT2, and NKT17 cells are largely tissue-resident. Parabiosis experiments showed that >95% of thymic iNKT cells remain host-derived after 30 days of shared circulation, and intra-thymic transfer demonstrates that mature NKT1 cells are rarely recovered in the spleen (9). Thus, thymic and peripheral effector pools are largely independent, and both derive from a distinct CCR7^+^ precursor population (**Figure 1**). Wang and Hogquist identified this precursor pool as PLZF^hi^ cells distinct from IL-4 producing NKT2 effectors. Within PLZF^hi^ iNKT cells, CCR7 and PD-1 discriminate precursors from effectors, with IL-4 production restricted to PD-1^+^ cells at steady state. CCR7^+^ precursors are highly proliferative and generate all three effector subsets following intra-thymic or intravenous transfer, indicating developmental potential both within and outside the thymus. In mixed bone marrow chimeras, Ccr7 deficient cells show impaired effector differentiation and defective localization to the thymic medulla, where IL-15 from medullary thymic epithelial cells promotes NKT1 commitment (24, 25). Direct RTE tracking using intra-thymic NHS-biotin labeling further confirmed that CCR7^+^ iNKT cells are enriched among cells actively exiting the thymus and display immature phenotypes (9). Egress of these cells depends on KLF2, which is selectively expressed in CCR7+ precursors and RTEs (9). In mixed bone marrow chimeras, Klf2 deficiency leads to thymic accumulation and marked depletion of RTEs, consistent with KLF2-driven egress via transcriptional activation of S1pr1 and Ccr7 (26).

Single-cell analyses further refined this model by identifying an RTE-like population positioned between thymic precursors and mature peripheral iNKT cells. These cells express trafficking and egress-associated genes (Ccr7, S1pr1, Sell, Klf2) while lacking strong NKT1, NKT2, or NKT17 programs. Upon entering peripheral tissues, they progressively acquire residency-associated genes (Atf3, Cd244, Cd69, Fos, Jun) and downregulate circulatory signatures (14). Subclustering revealed early tissue-specific divergence, with distinct signatures in spleen (Lgals1, S100a6), liver (Irf8, Xcl1), and lymph node (Dapl1, Sell, Tsc22d3), indicating that local cues begin shaping iNKT identity shortly after seeding (14). The importance of KLF2 in this process is further supported by conditional knockout studies: Klf2 loss blocks early thymic development (stages 1-2), reduces CCR7 expression, and severely depletes peripheral iNKT cells in spleen, liver, and lung, while sparing lymph node populations, suggesting partially KLF2-independent seeding mechanisms. An unresolved question is whether a cytotoxic Gzma/Ccl5-expressing cluster that retains high Klf2 and circulatory features represents a transitional RTE population rather than a long-lived NKT1 subset. Its proximity to the RTE cluster in UMAP supports this possibility, although its developmental origin remains unclear (14).

## Peripheral remodeling: tissue and antigen shape iNKT cell states

Single-cell analyses reveal that iNKT cells are extensively remodeled after thymic exit, shaped by tissue specific cues and antigen exposure (**Figure 1**).

### Tissue-specific effects on subset divergence

Wang et al. provided a systematic single-cell map of peripheral iNKT cells across liver, spleen, and lymph node, integrated with thymic populations (14). NKT1 cells show pronounced tissue-dependent divergence, forming largely distinct clusters across organs with low inter-organ correlation, whereas NKT17 cells converge into a single, highly correlated cluster, indicating relative resistance to local environmental cues. NKT1 cells in different tissues exhibit context-specific features, including enhanced survival and signaling pathways in the liver and stronger residency and activation signatures in lymph nodes, while maintaining more circulatory characteristics in spleen and liver (14). These findings extend earlier bulk RNA-seq work by Murray et al. (27), which showed that iNKT cells primarily cluster by subset identity rather than tissue of origin, indicating that core lineage programs are preserved despite tissue-driven remodeling. In addition, an interferon-stimulated gene (ISG)-high population is conserved across peripheral organs, with strong inter-organ similarity and evidence of conservation in humans (12, 14), suggesting that type I interferon signaling defines a tissue-independent transcriptional state. Although human tissue scRNAseq datasets remain limited, earlier studies demonstrated that fetal intestinal iNKT cells acquire a more differentiated phenotype than splenic or lymph node iNKT cells, suggesting that tissue-specific maturation is not unique to mice (16).

### Antigen-driven remodeling and the emergence of regulatory and memory-like states

Antigen exposure is a major driver of peripheral iNKT cell diversification, generating activation states absent from the thymus. Using longitudinal scRNAseq of splenic and adipose iNKT cells following αGalCer stimulation, Kane et al. defined the transcriptional dynamics of iNKT activation and differentiation (28). Early activation (4 hours) is characterized by rapid induction of cytokine programs, PLZF-associated genes, and metabolic pathways linked to glycolysis and biosynthesis, while canonical NKT1 and NKT17 signatures are transiently suppressed. Notably, this early activation program is broadly conserved across NKT1, NKT2, and NKT17 subsets, suggesting that antigen-driven responses overlay rather than erase lineage-associated programs. By 72 hours, cells transition toward proliferative and memory-like states marked by expression of Tcf7, Slamf6, Ccr7, Klrg1, Itga4, and Cxcr5. Subset-specific metabolic differences also emerge, with NKT2 and NKT17 cells exhibiting greater oxidative phosphorylation signatures and mitochondrial activity than NKT1 cells. Consistent with this, oxidative phosphorylation inhibition preferentially impairs IL-4, IL-13, and IL-17A production while sparing IFNγ, highlighting distinct metabolic requirements among activated subsets (28). Adipose iNKT cells display a distinct chronically activated phenotype even at steady state, characterized by elevated Nr4a genes, Nfat family members, Cd69, Il2, Myc, Klrg1, and Itga4 expression. Following a single αGalCer challenge, adipose iNKT cells maintain prolonged cytokine expression at 72 hours, a ‘smoldering’ activation pattern not observed in splenic cells, consistent with chronic endogenous activation at steady state. Interestingly, Il10 expression in adipose tissue is restricted primarily to NKT1-like cells, suggesting that NKT10 cells arise preferentially from the NKT1 lineage in this setting (28).

At 4 weeks post-stimulation, two induced populations emerge across multiple tissues. A KLRG1^+^ population display cytotoxic effector features resembling CD8 effector memory and NK cells, including expression of Gzmb, Gzma, Klrd1, Cx3cr1, and Zeb2 (29, 30). In contrast, a cMAF^+^ population exhibit transcriptional features associated with memory, precursor-exhausted, and Tr1-like states, including Il27ra, Ctla4, Lag3, Hif1a, and Maf (31, 32). These populations are mutually exclusive and transcriptionally more similar to NKT1 than to NKT2 or NKT17 cells (28). Consistent with this, IL-10-producing cMAF^+^ cells and granzyme-expressing KLRG1^+^ cells both co-produced IFNγ, whereas IL-17A and IL-10 expression remained mutually exclusive. Gene regulatory network analysis identified cMAF as the transcription factor most strongly associated with Il10 expression, supporting a central role in the regulatory iNKT program (28). Although cMAF^+^ cells share substantial transcriptional overlap with NKTfh cells, including expression of Tox, Slamf6, Tcf7, Il21, and Lag3, they rarely expressed CXCR5 or PD-1 and lacked BCL6, leading the authors to classify them as distinct from NKTfh cells. Notably, small populations of cMAF^+^ and KLRG1^+^ cells are already present in adipose tissue at steady state, consistent with chronic endogenous activation. Repeated αGalCer stimulation similarly induces adipose-like regulatory programs in splenic iNKT cells, including Tr1-associated signatures and reduced responsiveness to restimulation, suggesting that chronic activation converges on a shared regulatory iNKT cell state (28, 33).

### Human peripheral iNKT diversity

Compared with murine iNKT cells, human peripheral iNKT cells are less consistent with the classic NKT1, NKT2 and NKT17 framework (**Figure 1**). Early phenotypic studies identified functionally distinct CD4+, DN, and CD8+ iNKT cell populations and suggested a maturation trajectory characterized by loss of CD4, CCR7, and CD62L, acquisition of NK associated receptors including CD161, CD56, and NKG2A, and enhanced IFNg expression (16, 34–36). Studies of fetal human tissues showed that iNKT cells accumulated preferentially in the fetal small intestine, where they gain more differentiated phenotype and less proliferative capacity as compared to iNKT cells in fetal spleen and mesenteric lymph nodes (16). Notably, these changes occurred before establishment of the commensal microbiota, suggesting that tissue specific factors and endogenous CD1d ligands can shape iNKT cell state following peripheral seeding.

Recent scRNAseq studies have substantially refined this framework. Analysis of peripheral blood iNKT cells had identified precursor-like CD62L+LEF1+ populations, cytotoxic GZMB+GNLY+ populations, and activation associated states (37). Integrated analyses of thymus, cord blood, peripheral blood, and bone marrow revealed that human iNKT cells go through a trajectory of precursor, transitional, and effector states (12). Precursor populations are enriched for developmental regulators including SOX4, LEF1, TOX2, and ITM2A, whereas differentiated populations acquire a dominant Th1/17/NK-like effector program co-expressing TBX21 and RORC, together with GZMK, GZMA, KLRB1, GNLY, CCL5, CXCR4, IL18R1, and IL18RAP, indicating readiness for rapid cytokine driven responses (12, 15). Notably, GZMK rather than GZMB predominates in human effector iNKT and other Tinn cells, whereas mouse Tinn cells rely on pre-formed cytokine transcripts for rapid responses, suggestive of a distinct effector mechanism (12, 15).

Regulatory network analyses support a progressive differentiation model. SOX4 activity is highest in precursor populations and declines along pseudotime, whereas EOMES activity increases in differentiated cells and is associated with expression of GZMK, NKG7, KLRG1, CCL4 and LAG3+. Together with enrichment of RORA associated programs (15), these findings suggest that human iNKT cell differentiation relies on a transcriptional network that only partially overlaps with the Tbet/RORgt controlled programs in mice. Additional human regulons including MYBL1, CEBPD and FOSL2, further support the existence of species-specific regulatory circuitry within the Tinn compartment (15).

Two additional populations have emerged from recent analyses. First, a small subset of terminally differentiated DN iNKT cells displays a terminal effector memory RA-positive (TEMRA)-like phenotype characterized by re-expression of CD45RA, loss of CCR7, and enrichment of cytotoxic genes including GNLY and KLRG1 (12). These cells resemble conventional CD8+ TEMRA cells and likely represent a highly differentiated effector state that is not apparent in the thymus (38). Second, human CD8+ iNKT cells resolve into both precursor-like and effector populations (12). Notably, most peripheral CD8+ iNKT cells express CD8aa homodimers rather than CD8ab heterodimers, linking them transcriptionally to other innate-like lymphocyte populations rather than conventional cytotoxic T cells (39). Across these studies, CD4 and CD8 expressions showed only partial correspondence with transcriptional identity, indicating that coreceptor-based classification incompletely captures the functional diversity of human iNKT cells.

Together, current evidence supports a model in which human peripheral iNKT cells are organized along developmental and activation spectrum rather than the discrete effector lineages described in mice. The dominant effector state combines type 1, type 17, and NK-associated features, while additional populations such as TEMRA-like and CD8aa+ cells further expand the functional diversity of the human iNKT cell compartment (**Figure 1**). These species-specific features should be considered when translating insights from murine models to human iNKT cell immunotherapies.

## Discussion

scRNAseq studies reviewed here support a model that iNKT cell states are dynamic and context-dependent, influenced by developmental stage, tissue microenvironment, antigen experience, and species-specific constraints. While mouse models have provided fundamental insights into distinct lineage commitment, human data reveal a more gradual and plastic process that only partially overlaps with the murine paradigm.

In mice, thymic iNKT cells rapidly differentiate into distinct NKT1, NKT2 and NKT17 subsets that largely retain core identifies in the periphery. Yet, recent scRNAsesq data highlight NKT2-like states occupy intermediate positions along trajectories toward NKT1 and NKT17 fates, and antigen driven remodeling generates long-lived peripheral subsets without direct thymic counterparts, including regulatory NKT10 cells, cytotoxic KLRG1+ population, and NKTfh cells (14, 22, 23, 27, 28, 33, 40–43). Tissue specific remodeling is evident, particularly within the NKT1 cells, while NKT17 cells remain more conserved across organs (14). These findings reconcile bulk RNAseq data emphasizing subset dominant signatures with single cell resolution of intra-subset heterogeneity (27).

Human iNKT cells exhibit a more progressive maturation trajectory than their murine counterparts, with evidence that both developmental stage and aging influence the composition of the iNKT cell compartment. In the postnatal thymus, most iNKT cells display a naïve/precursor-like state resembling developing conventional CD4+ thymocytes, with only a minority acquiring a mixed type 1/17 effector signature (12, 15). Fetal iNKT cells already show tissue specific functional maturation, with striking accumulation, CD161 upregulation, and robust IFNg production in the small intestine independent of commensal flora (16). Cord blood enriches transitional populations with high PLZF and precursor features, while adult peripheral blood and bone marrow progressively favor CD4- Th1/17/NK-like effectors characterized by GZMK-biased cytotoxicity and innate cytokine responsiveness (12, 15). In parallel, murine analyses reveal that aging remodels subset homeostasis with progressive contraction of the NKT2 compartment (4), without fundamentally altering the core iNKT cell subset programs (44), though specific functional adaptations do emerge, including loss of granzyme A expression in NKT1 cells, upregulation of CD25 in NKT17 cells, and decreased proliferation (44). In humans, aging is associated with a striking decline in circulating iNKT cell frequency accompanied by a shift toward higher CD4+ subset, lower DN subset, and a skewing toward Th2 biased cytokine production (45). Antigen driven remodeling is supported by direct stimulation experiments showing rapid induction of distinct cytokine-producing, cytotoxic, and potential regulatory states (37), as well as by progressive effector enrichment from cord blood to adult blood/BM, especially the identification of a CD45RA+ CCR7- TEMRA-like subset in peripheral effectors (12, 15), typically associated with chronic repeated antigen exposure in conventional T cells (46).

Cross species comparisons reveal both conserved and divergent features. Core programs (PLZF-driven innateness, effector capabilities) are shared (12, 13, 15), yet humans lack a prominent type 2 compartment, exhibit greater polyfunctionality and plasticity, rely on distinct regulatory network (eg. prominent EOMES, CEBPD, MYBL1, FOSL2 and declining SOX4 along pseudotime) and display unique populations such as CD8aa subsets (both naïve-like and effector) and TEMRA-like states (12, 13, 15). The reduced thymic diversification in humans and pigs relative to mice suggests that substantial functional specialization occurs progressively later in development across species (12, 19). These differences caution against direct extrapolation from mouse models and emphasize the value of human centric systems for mechanistic insight.

Despite major advances, important questions remain, including the molecular basis of tissue specific remodeling, the developmental relationships between regulatory and helper-like states, and how age and environment driven transitions influence disease susceptibility. Nevertheless, the emerging view of iNKT cells as dynamic transcriptional states has clear therapeutic implications. Rather than targeting fixed subsets, future strategies may seek to direct human iNKT cells toward desired cytotoxic, regulatory, or helper programs by manipulating the transcriptional and epigenetic networks controlling state transition *in vivo*.

## References

[1] Kronenberg M . Toward an understanding of NKT cell biology: progress and paradoxes. Annu Rev Immunol. (2005) 23:877–900. doi: 10.1146/annurev.immunol.23.021704.115742 15771592

[2] Niedzielska M Chalmers A Popis MC Altman-Sharoni E Addis S Beulen R . CAR-iNKT cells: redefining the frontiers of cellular immunotherapy. Front Immunol. (2025) 16:1625426. doi: 10.3389/fimmu.2025.1625426 40718496 PMC12291068

[3] Benlagha K Wei DG Veiga J Teyton L Bendelac A . Characterization of the early stages of thymic NKT cell development. J Exp Med. (2005) 202:485–92. doi: 10.1084/jem.20050456 16087715 PMC2212852

[4] Lee YJ Holzapfel KL Zhu J Jameson SC Hogquist KA . Steady-state production of IL-4 modulates immunity in mouse strains and is determined by lineage diversity of iNKT cells. Nat Immunol. (2013) 14:1146–54. doi: 10.1038/ni.2731 24097110 PMC3824254

[5] Pellicci DG Hammond KJ Uldrich AP Baxter AG Smyth MJ Godfrey DI . A natural killer T (NKT) cell developmental pathway involving a thymus-dependent NK1.1(-)CD4(+) CD1d-dependent precursor stage. J Exp Med. (2002) 195:835–44. doi: 10.1084/jem.20011544 11927628 PMC2193721

[6] Baranek T de Amat Herbozo C Mallevaey T Paget C . Deconstructing iNKT cell development at single-cell resolution. Trends Immunol. (2022) 43:503–12. doi: 10.1016/j.it.2022.04.012 35654639

[7] Crosby CM Kronenberg M . Tissue-specific functions of invariant natural killer T cells. Nat Rev Immunol. (2018) 18:559–74. doi: 10.1038/s41577-018-0034-2 29967365 PMC6343475

[8] Lee YJ Wang H Starrett GJ Phuong V Jameson SC Hogquist KA . Tissue-specific distribution of iNKT cells impacts their cytokine response. Immunity. (2015) 43:566–78. doi: 10.1016/j.immuni.2015.06.025 26362265 PMC4575275

[9] Wang H Hogquist KA . CCR7 defines a precursor for murine iNKT cells in thymus and periphery. eLife. (2018) 7:e34793. doi: 10.7554/elife.34793 30102153 PMC6115192

[10] Engel I Seumois G Chavez L Samaniego-Castruita D White B Chawla A . Innate-like functions of natural killer T cell subsets result from highly divergent gene programs. Nat Immunol. (2016) 17:728–39. doi: 10.1038/ni.3437 27089380 PMC4944658

[11] Krovi SH Loh L Spengler A Brunetti T Gapin L . Current insights in mouse iNKT and MAIT cell development using single cell transcriptomics data. Semin Immunol. (2022) 60:101658. doi: 10.1016/j.smim.2022.101658 36182863 PMC11854848

[12] Jayasinghe RG Hollingsworth D Schedler NC Landy E Boonchalermvichian C Gupta B . Single-cell transcriptomic profiling reveals diversity in human iNKT cells across hematologic tissues. Cell Rep. (2025) 44:115587. doi: 10.1016/j.celrep.2025.115587 40305288 PMC12255272

[13] Maas-Bauer K Kohler N Stell AV Zwick M Acharya S Rensing-Ehl A . Single-cell transcriptomics reveal different maturation stages and sublineage commitment of human thymic invariant natural killer T cells. J Leukoc Biol. (2024) 115:401–9. doi: 10.1093/jleuko/qiad113 37742056 PMC10799303

[14] Wang J Loveless I Adrianto I Liu T Subedi K Wu X . Single-cell analysis reveals differences among iNKT cells colonizing peripheral organs and identifies Klf2 as a key gene for iNKT emigration. Cell Discov. (2022) 8:75. doi: 10.1038/s41421-022-00432-z 35915069 PMC9343440

[15] Loh L Carcy S Krovi HS Domenico J Spengler A Lin Y . Unraveling the phenotypic states of human innate-like T cells: comparative insights with conventional T cells and mouse models. Cell Rep. (2024) 43:114705. doi: 10.1101/2023.12.07.570707 39264810 PMC11552652

[16] Loh L Ivarsson MA Michaelsson J Sandberg JK Nixon DF . Invariant natural killer T cells developing in the human fetus accumulate and mature in the small intestine. Mucosal Immunol. (2014) 7:1233–43. doi: 10.1038/mi.2014.13 24646938

[17] Wang J Adrianto I Subedi K Liu T Wu X Yi Q . Integrative scATAC-seq and scRNA-seq analyses map thymic iNKT cell development and identify Cbfbeta for its commitment. Cell Discov. (2023) 9:61. doi: 10.1038/s41421-023-00547-x 37336875 PMC10279728

[18] Dashtsoodol N Shigeura T Aihara M Ozawa R Kojo S Harada M . Alternative pathway for the development of V(alpha)14(+) NKT cells directly from CD4(-)CD8(-) thymocytes that bypasses the CD4(+)CD8(+) stage. Nat Immunol. (2017) 18:274–82. doi: 10.1038/ni.3668 28135253

[19] Gu W Madrid DMC Joyce S Driver JP . A single-cell analysis of thymopoiesis and thymic iNKT cell development in pigs. Cell Rep. (2022) 40:111050. doi: 10.1016/j.celrep.2022.111050 35793622 PMC9704770

[20] De Calisto J Wang N Wang G Yigit B Engel P Terhorst C . SAP-dependent and -independent regulation of innate T cell development involving SLAMF receptors. Front Immunol. (2014) 5:186. doi: 10.3389/fimmu.2014.00186 24795728 PMC4005954

[21] Constantinides MG Bendelac A . Transcriptional regulation of the NKT cell lineage. Curr Opin Immunol. (2013) 25:161–7. doi: 10.1016/j.coi.2013.01.003 23402834 PMC3647452

[22] Harsha Krovi S Zhang J Michaels-Foster MJ Brunetti T Loh L Scott-Browne J . Thymic iNKT single cell analyses unmask the common developmental program of mouse innate T cells. Nat Commun. (2020) 11:6238. doi: 10.1038/s41467-020-20073-8 33288744 PMC7721697

[23] Baranek T Lebrigand K de Amat Herbozo C Gonzalez L Bogard G Dietrich C . High dimensional single-cell analysis reveals iNKT cell developmental trajectories and effector fate decision. Cell Rep. (2020) 32:108116. doi: 10.1016/j.celrep.2020.108116 32905761

[24] White AJ Jenkinson WE Cowan JE Parnell SM Bacon A Jones ND . An essential role for medullary thymic epithelial cells during the intrathymic development of invariant NKT cells. J Immunol. (2014) 192:2659–66. doi: 10.4049/jimmunol.1303057 24510964 PMC3948113

[25] Cui G Hara T Simmons S Wagatsuma K Abe A Miyachi H . Characterization of the IL-15 niche in primary and secondary lymphoid organs *in vivo*. Proc Natl Acad Sci USA. (2014) 111:1915–20. doi: 10.1073/pnas.1318281111 24449915 PMC3918838

[26] Carlson CM Endrizzi BT Wu J Ding X Weinreich MA Walsh ER . Kruppel-like factor 2 regulates thymocyte and T-cell migration. Nature. (2006) 442:299–302. doi: 10.1038/nature04882 16855590

[27] Murray MP Engel I Seumois G Herrera-De la Mata S Rosales SL Sethi A . Transcriptome and chromatin landscape of iNKT cells are shaped by subset differentiation and antigen exposure. Nat Commun. (2021) 12:1446. doi: 10.1038/s41467-021-21574-w 33664261 PMC7933435

[28] Kane H LaMarche NM Ni Scannail A Garza AE Koay HF Azad AI . Longitudinal analysis of invariant natural killer T cell activation reveals a cMAF-associated transcriptional state of NKT10 cells. eLife. (2022) 11:e76586. doi: 10.7554/elife.76586 36458691 PMC9831610

[29] Omilusik KD Best JA Yu B Goossens S Weidemann A Nguyen JV . Transcriptional repressor ZEB2 promotes terminal differentiation of CD8+ effector and memory T cell populations during infection. J Exp Med. (2015) 212:2027–39. doi: 10.1038/ncomms9306 26503445 PMC4647262

[30] Bottcher JP Beyer M Meissner F Abdullah Z Sander J Hochst B . Functional classification of memory CD8(+) T cells by CX3CR1 expression. Nat Commun. (2015) 6:8306. 26404698 10.1038/ncomms9306 PMC4667439

[31] Chu T Wu M Hoellbacher B de Almeida GP Wurmser C Berner J . Precursors of exhausted T cells are pre-emptively formed in acute infection. Nature. (2025) 640:782–92. doi: 10.1038/s41586-024-08451-4 39778709 PMC12003159

[32] Pot C Jin H Awasthi A Liu SM Lai CY Madan R . Cutting edge: IL-27 induces the transcription factor c-Maf, cytokine IL-21, and the costimulatory receptor ICOS that coordinately act together to promote differentiation of IL-10-producing Tr1 cells. J Immunol. (2009) 183:797–801. doi: 10.4049/jimmunol.0901233 19570826 PMC2768608

[33] Sag D Krause P Hedrick CC Kronenberg M Wingender G . IL-10-producing NKT10 cells are a distinct regulatory invariant NKT cell subset. J Clin Invest. (2014) 124:3725–40. doi: 10.1172/jci72308 25061873 PMC4151203

[34] Gumperz JE Miyake S Yamamura T Brenner MB . Functionally distinct subsets of CD1d-restricted natural killer T cells revealed by CD1d tetramer staining. J Exp Med. (2002) 195:625–36. doi: 10.1084/jem.20011786 11877485 PMC2193772

[35] Sandberg JK Bhardwaj N Nixon DF . Dominant effector memory characteristics, capacity for dynamic adaptive expansion, and sex bias in the innate Valpha24 NKT cell compartment. Eur J Immunol. (2003) 33:588–96. doi: 10.1002/eji.200323707 12616479

[36] Eger KA Sundrud MS Motsinger AA Tseng M Van Kaer L Unutmaz D . Human natural killer T cells are heterogeneous in their capacity to reprogram their effector functions. PloS One. (2006) 1:e50. doi: 10.1371/journal.pone.0000050 17183680 PMC1762372

[37] Zhou L Adrianto I Wang J Wu X Datta I Mi QS . Single-cell RNA-seq analysis uncovers distinct functional human NKT cell sub-populations in peripheral blood. Front Cell Dev Biol. (2020) 8:384. doi: 10.3389/fcell.2020.00384 32528956 PMC7264113

[38] Muroyama Y Wherry EJ . Memory T-cell heterogeneity and terminology. Cold Spring Harb Perspect Biol. (2021) 13(10):a037929. doi: 10.1101/cshperspect.a037929 33782027 PMC8485749

[39] Vandereyken M James OJ Swamy M . Mechanisms of activation of innate-like intraepithelial T lymphocytes. Mucosal Immunol. (2020) 13:721–31. doi: 10.1038/s41385-020-0294-6 32415229 PMC7434593

[40] Shimizu K Sato Y Shinga J Watanabe T Endo T Asakura M . KLRG+ invariant natural killer T cells are long-lived effectors. Proc Natl Acad Sci USA. (2014) 111:12474–9. doi: 10.1073/pnas.1406240111 25118276 PMC4151776

[41] King IL Fortier A Tighe M Dibble J Watts GF Veerapen N . Invariant natural killer T cells direct B cell responses to cognate lipid antigen in an IL-21-dependent manner. Nat Immunol. (2011) 13:44–50. doi: 10.1038/ni.2172 22120118 PMC3833037

[42] Chang PP Barral P Fitch J Pratama A Ma CS Kallies A . Identification of Bcl-6-dependent follicular helper NKT cells that provide cognate help for B cell responses. Nat Immunol. (2011) 13:35–43. doi: 10.1038/ni.2166 22120117

[43] Tonti E Fedeli M Napolitano A Iannacone M von Andrian UH Guidotti LG . Follicular helper NKT cells induce limited B cell responses and germinal center formation in the absence of CD4(+) T cell help. J Immunol. (2012) 188:3217–22. doi: 10.4049/jimmunol.1103501 22379027 PMC3559029

[44] Papadogianni G Ravens I Dittrich-Breiholz O Bernhardt G Georgiev H . Impact of aging on the phenotype of invariant natural killer T cells in mouse thymus. Front Immunol. (2020) 11:575764. doi: 10.3389/fimmu.2020.575764 33193368 PMC7662090

[45] Jing Y Gravenstein S Chaganty NR Chen N Lyerly KH Joyce S . Aging is associated with a rapid decline in frequency, alterations in subset composition, and enhanced Th2 response in CD1d-restricted NKT cells from human peripheral blood. Exp Gerontol. (2007) 42:719–32. doi: 10.1016/j.exger.2007.01.009 17368996

[46] Sallusto F Geginat J Lanzavecchia A . Central memory and effector memory T cell subsets: function, generation, and maintenance. Annu Rev Immunol. (2004) 22:745–63. doi: 10.1146/annurev.immunol.22.012703.104702 15032595

